# Percutaneous Transhepatic Direct Portosystemic Shunt for a Patient With Budd-Chiari Syndrome Using a Balloon as a Target in a Stenotic Inferior Vena Cava

**DOI:** 10.7759/cureus.44967

**Published:** 2023-09-09

**Authors:** Kisako Fujiwara, Takayuki Kondo, Kentaro Fujimoto, Jun Koizumi, Naoya Kato

**Affiliations:** 1 Gastroenterology, Chiba University, Chiba, JPN; 2 Radiology, Chiba University, Chiba, JPN

**Keywords:** portal hypertension, inferior vena cava, budd-chiari syndrome, transjugular intrahepatic portosystemic shunt, direct intrahepatic portosystemic shunt

## Abstract

Budd-Chiari syndrome (BCS) patients with portal hypertension are often treated with a direct intrahepatic portosystemic shunt (DIPS) or transjugular intrahepatic portosystemic shunt (TIPS) and angioplasty. DIPS can be problematic, however, due to the technical difficulty of the procedure. To address this problem, we describe a method using the balloon used for inferior vena cava (IVC) dilatation as a puncture target to safely perform DIPS in a BCS patient with complete hepatic vein occlusion and stenosis of the IVC. To perform balloon dilation, the puncture is made through the internal jugular vein, and the guidewire is advanced to the IVC with stenosis. After dilatation of the IVC, the direct left lateral subdistrict branch of the portal vein is percutaneously punctured directly from the cardiac fossa (targeting the inflated balloon in the IVC), and the IVC puncture is done through the portal vein. After creating a pull-through route, a stent is placed between the left portal vein and the IVC. The procedure is completed without any complications. This technique has the potential to form the basis of a safe and reliable DIPS procedure.

## Introduction

A direct intrahepatic portosystemic shunt (DIPS) can be inserted either via the intravascular route or percutaneous route, which is a modification of the transjugular intrahepatic portosystemic shunt (TIPS). This procedure has been reported as an effective treatment for Budd-Chiari syndrome (BCS) [1]. However, inserting DIPS via the intravascular route can sometimes be difficult because the structure of the Rösch-Uchida Transjugular Liver Access Set (Cook Medical, Tokyo, Japan) limits the puncture angle. Conversely, inserting a DIPS via the percutaneous route can help overcome this limitation. Nevertheless, there is a restriction on the puncture route when using the percutaneous approach. In particular, the stenotic inferior vena cava (IVC) is difficult to visualize with ultrasound, increasing the risk of intra-abdominal bleeding due to IVC perforation. To address this issue, we have successfully and safely performed a DIPS inserted via the percutaneous route with a balloon-targeted puncture in a case of BCS.

This method is effective for IVC punctures because the implanted balloon in the IVC dilates the stenotic area and also serves as a puncture marker. To the best of our knowledge, this is the first report on the usefulness of balloon-targeted puncture during DIPS procedure inserted via the percutaneous route, although there has been a previous report on TIPS using a balloon placed in the portal vein [2].

## Case presentation

**Figure 1:**
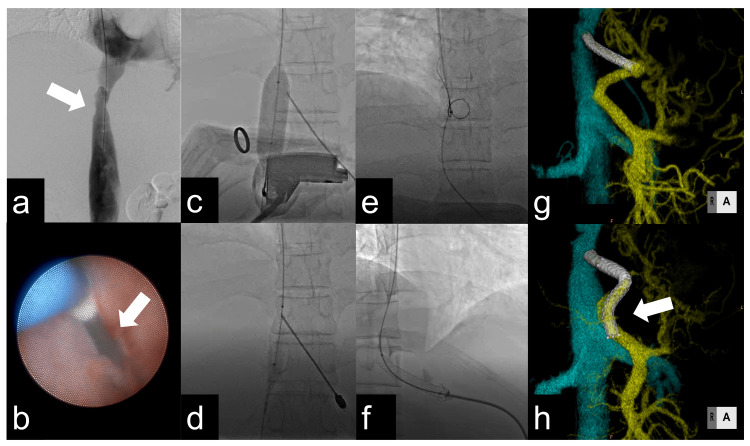
Reference images before and after treatment (a) Angiographic image (arrows mark the subhepatic vena cava stenosis); (b) vascular endoscopy image (arrow marks the membranous stenosis of the hepatic vein); (c) the IVC was punctured through the portal vein with the dilated balloon as the target; (d) we confirmed that the needle had reached the IVC with the inflated balloon ruptured by the puncture; (e) the guidewire was passed from the portal vein to the IVC, caught with a snare catheter, and guided to the internal jugular vein; (f) a stent was placed in the pathway connecting the portal vein to the IVC; (g, h) an additional stent (arrow) was placed in the main trunk of the portal vein, resulting in a looser angle of the stent than before and less obstruction to internal blood flow.

A 47-year-old man visited our hospital because of liver dysfunction and edema in the past four months. The patient was diagnosed with BCS of unknown cause. Liver transplantation was not indicated due to a lack of donor candidates. On admission, the patient’s Child-Pugh score was 12, and the model for end-stage liver disease (MELD) score was 12. A triphasic computed tomography (CT) scan showed hepatic parenchymal heterogeneity with massive pleuroperitoneal effusion, complete occlusion of the hepatic veins, and a stenotic subhepatic vena cava. Venography and angioscopy were also performed to confirm these findings (Figure 1a).

During venography, the guidewire could not be placed in the hepatic vein. Angioscopy (FiberTech, Sakura, Japan) showed membranous stenosis in the hepatic IVC (Figure 1b). Stenting from the IVC to the portal vein was difficult due to angulation problems between the IVC and portal vein. Therefore, we chose percutaneous DIPS instead of transjugular DIPS.

The right internal jugular vein was punctured, and a 0.035-inch 180-cm Radifocus Guidewire (Terumo, Tokyo, Japan) was inserted into a 12-Fr 25 cm sheath (Medikit, Tokyo, Japan), and a Maxi-LDTM PTA balloon catheter (Cordis, Tokyo, Japan) was introduced to the level of the renal vein bifurcation. The IVC pressure was 17 mmHg, and near the right atrium, the pressure was 2 mmHg. The IVC pressure was reduced to 9 mmHg after dilation of the membranous stenosis of the IVC with a balloon.

We percutaneously punctured the umbilical portion of the origin of the left lateral subdistrict branch of the portal vein (P3) under ultrasound guidance using an 18G 20-cm elastic needle. After ultrasound visibility became obscured, the needle was advanced to the inflated IVC balloon as a target under fluoroscopy, and it was confirmed that the IVC was punctured when the balloon ruptured (Figures 1c-1d).

A 0.035-inch 180-cm Radifocus Guidewire (Terumo, Japan) was threaded through the cephalic side of the IVC and was captured with a snare catheter (ONE Snare, 20 mm; Merit Medical Systems, South Jordan, UT) inserted through the sheath of the internal jugular vein. The snare and wire were withdrawn through the transjugular vascular sheath (Figure 1e).

A 6-Fr 25 cm sheath (Medikit) was inserted over the wire from the pull-through route to the IVC, and a 5-Fr 110 cm Pigtail Marker Catheter (Medikit) was inserted. Portal vein angiography showed portal vein stenosis and reflux of the portal blood flow. The portal pressure was 35 mmHg, and the portosystemic gradient was 33 mmHg. After confirming the site of stenosis by digital subtraction angiography, a 7 mm x 57 mm Express LD stent (Boston Scientific, Marlborough, MA) was placed (Figure 1f). The stent was dilated to 7.5 mm, the portosystemic gradient decreased to 24 mmHg, and the improvement of in-stent blood flow was confirmed. The procedure was then completed with embolization of the liver puncture tract using 0.1 mL of 50% n-butyl-cyanoacrylate (NBCA).

Angiography performed two months after discharge showed severe stenosis in the stent. One of the causes of stenosis might be explained by the steep angle between the portal vein and the stent, which could have impaired blood flow in the stent. To treat this impairment, an additional 10 mm x 80 mm stent (Boston Scientific, Massachusetts, United States) protruding into the main portal vein was implanted. After additional stenting, no restenosis was observed, and ascites disappeared (Figures 1g-1h).

## Discussion

In this case, a patient with an obstruction in the IVC of the liver, as well as three branches of the hepatic vein, was successfully treated with percutaneous DIPS by targeting a balloon placed in the collapsed IVC. To the best of our knowledge, this is the first report of a DIPS inserted via the percutaneous route procedure using a dilated balloon in the IVC as a target for puncture.

The problem associated with the BCS treatment is that medical therapy alone, such as anticoagulation, may result in a poor prognosis [3]. TIPS has been reported to be an effective treatment as an invasive therapy [4,5]. However, TIPS is challenging to perform if the hepatic vein is obstructed or if the portal vein is located cephalad of the intrahepatic vein. DIPS, a direct short circuit between the IVC and portal vein, is also applicable to BCS patients with hepatic vein obstruction and caudal lobe enlargement.

The most difficult aspect of the DIPS technique is how to create a safe pathway between the IVC and portal vein. To solve this problem, in this report, a balloon was placed in the hepatic IVC, which was punctured using the inflated balloon as the target. For the accurate and safe puncturing of the IVC, this method is effective because it dilates the collapsed IVC and serves as a puncture marker.

The intracardiac echocardiography (ICE) approach is also reportedly a relatively safe method during the DIPS procedure [6]. In this case, ICE can evaluate how the puncture needle progresses from the portal vein. However, it is difficult to determine whether the needle penetrates the IVC, and it may cause a risk of vascular perforation.

Another problem with DIPS in BCS cases is that the angle of stent placement is likely to be steep, and there is a high possibility of hemodynamic disturbance [7]. In the present case, the stenting angle was also steep due to the transhepatic implant, resulting in stent stenosis two months after surgery. Therefore, an additional stent was placed to reach the main portal vein to reduce the stenting angle and improve the blood flow in the stent. Thereafter, no severe stent stenosis was observed.

## Conclusions

A balloon catheter used for angioplasty can also be used as a puncture target for DIPS to safely puncture the IVC in BCS patients with portal hypertension and stenosis of the IVC. This approach may facilitate DIPS in institutions with limited technical experience. Furthermore, another problem with DIPS is that the stent is planted at a steep angle, which may result in stenosis of the stent. Therefore, care should be taken with the angle of stent placement, and if necessary, additional stents should be considered.
